# *Smad3* Deficiency Ameliorates Hepatic Fibrogenesis through the Expression of Senescence Marker Protein-30, an Antioxidant-Related Protein

**DOI:** 10.3390/ijms141223700

**Published:** 2013-12-04

**Authors:** Da-Hee Jeong, Meeyul Hwang, Jin-Kyu Park, Moon-Jung Goo, Il-Hwa Hong, Mi-Ran Ki, Akihito Ishigami, Ah-Young Kim, Eun-Mi Lee, Eun-Joo Lee, Kyu-Shik Jeong

**Affiliations:** 1 Department of Pathology, College of Veterinary Medicine, Kyungpook National University, Daegu 702-701, Korea; E-Mails: daheej.kr@gmail.com (D.-H.J.); meeyulh@gmail.com (M.H.); 820jinkyu@hanmail.net (J.-K.P.); goomoonj@hotmail.com (M.-J.G.); ihhong@gnu.ac.kr (I.-H.H.); mrgee@hanmail.net (M.-R.K.); pretersensual@hanmail.net (A.-Y.K.); nikeun@hanmail.net (E.-M.L.); miffy525@hanmail.net (E.-J.L.); 2 Aging Regulation, Tokyo Metropolitan Institute of Gerontology, Tokyo 173-0015, Japan; E-Mail: ishigami@phar.toho-u.ac.jp

**Keywords:** liver fibrosis, Smad3, SMP-30, antioxidant

## Abstract

Smad3 is a key mediator of the transforming growth factor (TGF)-β1 signaling pathway that plays central role in inflammation and fibrosis. In present study, we evaluated the effect of Smad3 deficiency in *Smad3*^−/−^ mice with carbon tetrachloride (CCl_4_)-induced liver fibrosis. The animals were received CCl_4_ or olive oil three times a week for 4 weeks. Histopathological analyses were performed to evaluate the fibrosis development in the mice. Alteration of protein expression controlled by Smad3 was examined using a proteomic analysis. CCl_4_-induced liver fibrosis was rarely detected in *Smad3*^−/−^ mice compared to *Smad3*^+/+^. Proteomic analysis revealed that proteins related to antioxidant activities such as senescence marker protein-30 (*SMP30*), selenium-binding proteins (SP56) and glutathione *S*-transferases (GSTs) were up-regulated in *Smad3*^−/−^ mice. Western blot analysis confirmed that *SMP30* protein expression was increased in *Smad3*^−/−^ mice. And *SMP30* levels were decreased in CCl_4_-treated *Smad3*^+/+^ and *Smad3*^−/−^ mice. These results indicate that Smad3 deficiency influences the proteins level related to antioxidant activities during early liver fibrosis. Thus, we suggest that Smad3 deteriorate hepatic injury by inhibitor of antioxidant proteins as well as mediator of TGF-β1 signaling.

## 1. Introduction

Smad proteins are intracellular mediators that respond to transforming growth factor (TGF)-β, an important regulatory cytokine that affects the production, degradation, and accumulation of extracellular matrix proteins during the development of liver fibrosis [1]. Smad proteins are involved in intracellular signaling in response to TGF-β family members. Smad proteins are classified as receptor-regulated Smads (R-Smads), common mediator Smads (Co-Smads), or inhibitory Smads (I-Smads). R-Smads are directly phosphorylated by receptors and translocate to the nucleus where they bind to response elements and regulate gene expression [2]. Smad3 belongs to the R-Smads family. This factor is phosphorylated by receptors and translocates to the nucleus where it activates gene expression.

Impairment of the Smad pathway results in an escape from growth inhibition, thereby promoting cell proliferation and contributing to carcinogenesis [3]. In particular, Smad3 is a major player in signal transduction pathways associated with fibrogenesis [4]. And several fibrotic markers are attenuated in *Smad3* knockout (KO) mice, resulting in the hypothesis that directing Smad3 to a clinical target might inhibit fibrosis [5,6]. In addition, the radiation-induced expression of α-smooth muscle actin (α-SMA) in cutaneous fibroblasts of *Smad3* KO mice is reduced to 25% of that found in cells from *Smad3* wild-type (WT) mice [7,8]. When acute liver injury is induced by the administration of CCl_4_, *Smad3* KO mice show approximately one-half of the induction of hepatic collagen type I mRNA expression compared to *Smad3* wild type (WT) mice [9]. These results have led to the speculation that Smad3 signaling specifically mediates the fibrotic effects of TGF-β. But, previous studies of Smad3 targets in an animal model of liver injury did not reveal a clear-cut mechanism underlying such activity.

A hepatic proteomic analysis was also conducted to study thioacetamide-induced cirrhosis in rats [10]. However, no proteomic analysis has unequivocally determined the relationship between Smad3 and hepatic fibrogenesis.

Here, we investigated the influence of *Smad3* on liver fibrogenesis by measuring hepatic protein expression in *Smad3* KO mice after CCl_4_ treatment for 4 weeks. Histological and proteomic analyses were used to monitor changes of protein expression during the course of hepatic fibrogenesis in these mice.

## 2. Results

### 2.1. Fibrotic Changes in Livers after the Chronic Administration of CCl_4_

**Figure 1.**
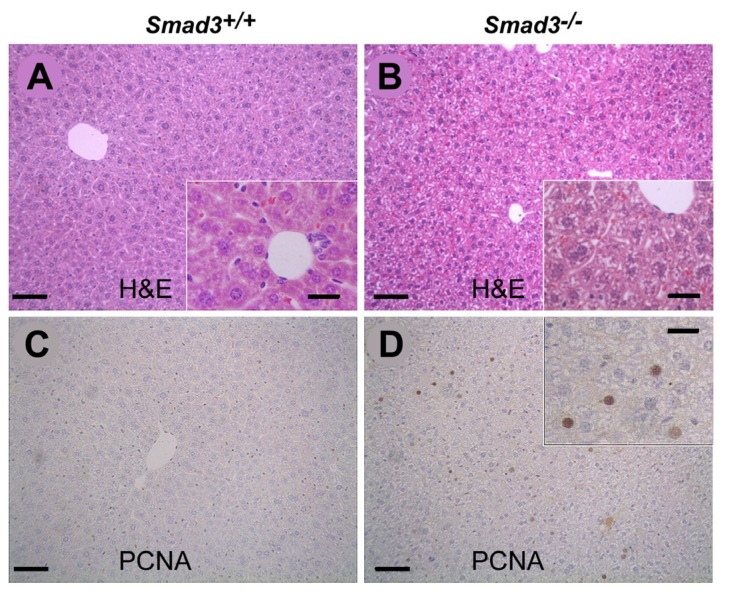
Histopathological analysis of livers from *Smad3*^+/+^ and *Smad3*^−/−^ mice. Liver tissues from *Smad3*^+/+^ (**A** and **C**) and *Smad3*^−/−^ (**B** and **D**) mice. Results for H & E staining (**A** and **B**) and immunohistochemistry for PCNA (**C** and **D**) are shown. Scale bars represent 50 μm. Inserts are high magnification fields from each figure. Scale bars represent 100 μm.

To evaluate the influence of Smad3 deficiency on hepatic fibrogenesis induced by repeated CCl_4_ injection, we first examined the H & E-stained livers of *Smad3* WT and *Smad3* KO mice (Figure 1A,B). Histological analysis revealed an increased hepatocyte population and loosening of the cell-to-cell tight junctions in the *Smad3* KO mice. When sections from the *Smad3* KO mice were assessed with immunohistochemical staining using an anti-PCNA antibody (Figure 1D), hepatocyte replication in the livers of *Smad3* KO mice was found to be markedly greater than that found in *Smad3* WT mice (Figure 1C).

**Figure 2.**
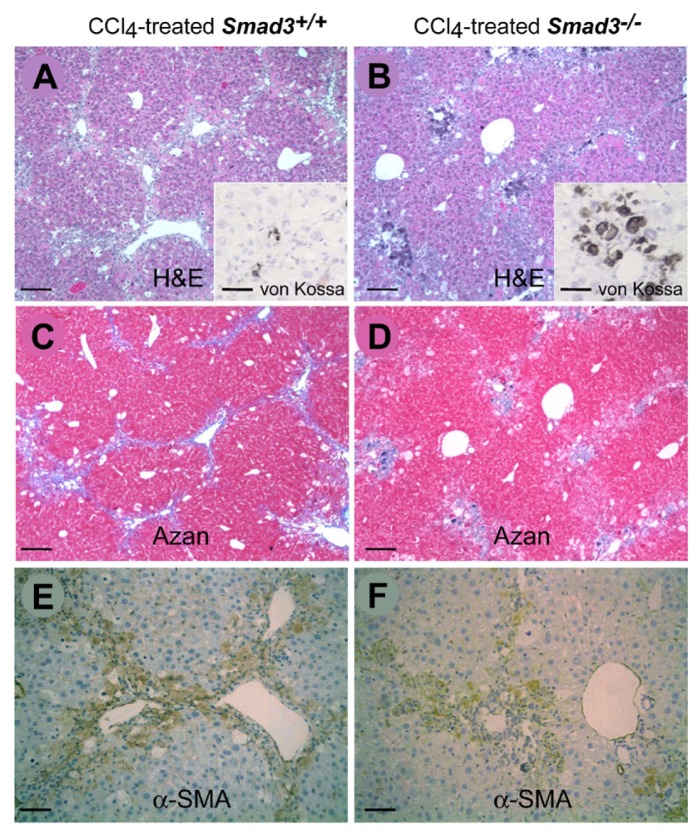
Hepatic fibrotic changes of Smad3 mutant mice after CCl_4_ treatment. Liver tissues of CCl_4_-treated *Smad3*^+/+^ (**A**, **C** and **E**) and *Smad3*^−/−^ (**B**, **D** and **F**) mice. H & E stain (**A** and **B**); azan stain (**C** and **D**); and immunostain for α-SMA (**E** and **F**). Scale bars represent 25 μm. Inserts (**A** and **B**) are von Kossa-stained samples of each figure. Scale bars represent 100 μm.

After four weeks of exposure to CCl_4_, liver fibrosis in the *Smad3* WT mice was more severe than in the *Smad3* KO mice (Figure 2 and Table 1). However, more calcium deposition was detected in the CCl_4_-treated *Smad3* KO mice compared to the CCl_4_-treated *Smad3* WT mice (inserts of Figure 2A,B). These findings were observed focally in the centrilobular region and destroyed hepatocytes of the CCl_4_-treated *Smad3* KO mice. The fibrotic changes were confirmed in liver tissues stained with azan (Figure 2C,D). Additionally, immunohistochemical analysis revealed that the expression of α-SMA in the livers of CCl_4_-treated *Smad3* WT mice was greater than that in the *Smad3* KO mice (Figure 2E,F and Table 1).

### 2.2. Analysis of Smad3-Related Proteins in Liver after the Chronic Administration of CCl_4_

**Figure 3.**
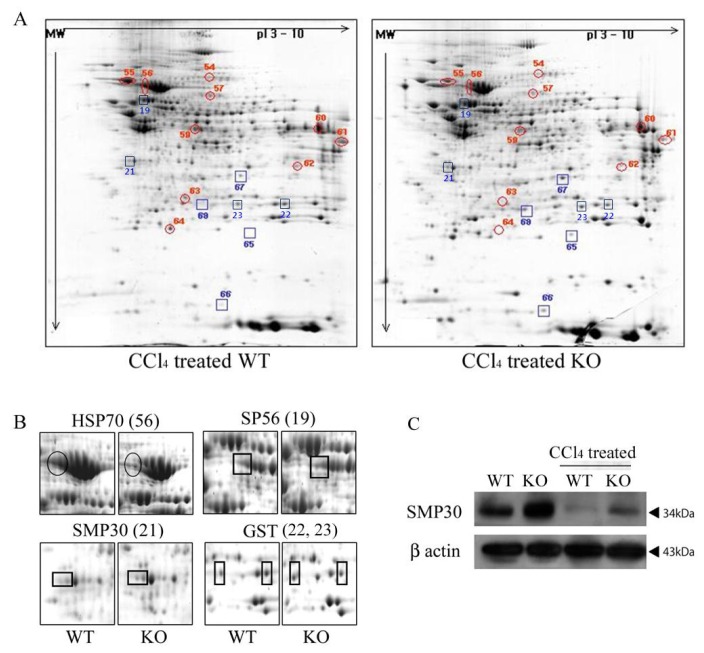
Substantial differentiated spots in liver tissues of *Smad3*^−/−^ mice. (**A**) Gel images of two dimensional gel electrophoresis (2-DE). Protein from liver tissue of *Smad3*^+/+^ and *Smad3*^−/−^ mice treated with CCl_4_ was separated on a pH 3–10 IPG strip in the first dimension and on an SDS-PAGE (12%) gel in the second dimension; (**B**) Clearly visible spots of the four proteins HSP70, *SMP30*, SP56 and GST are reproduced from gel images (SDS-PAGE) on a zoom-in view. WT: *Smad3*^+/+^ mice treated with CCl_4_; KO: *Smad3*^−/−^ mice treated with CCl_4_; and (**C**) Immunoblot analysis for *SMP30* and β-actin (as an internal standard).

**Figure 4.**
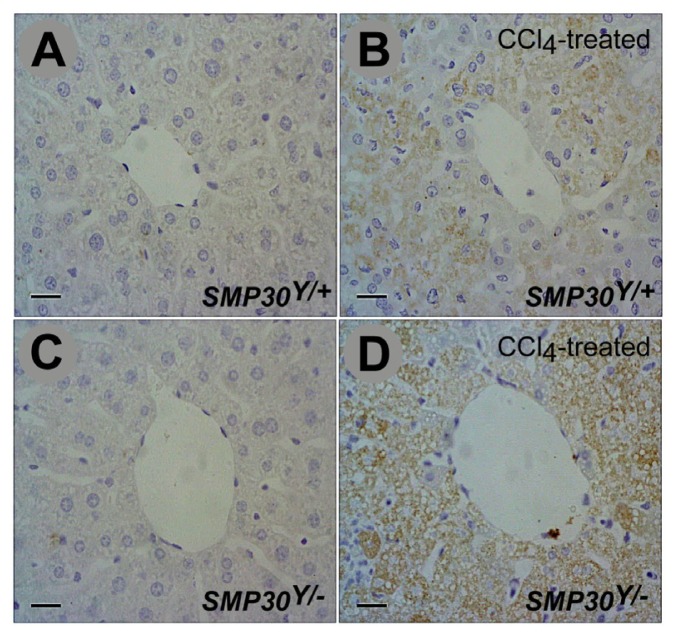
Expression of p-*Smad3* in the *SMP30^Y^*^/−^ mice. Immunohistopathological detection of p-*Smad3* in liver tissues from (**A**) *SMP30^Y^*^/+^; (**B**) CCl_4_-treated *SMP30^Y^*^/+^; (**C**) *SMP30^Y^*^/−^; and (**D**) CCl_4_-treated *SMP30^Y^*^/−^ mice. Cont indicates the non-treated control. Expression of p-*Smad3* in the CCl_4_-treated liver significantly increased around the central vein in *SMP30^Y^*^/−^ mice (**D**) compared to *SMP30^Y^*^/+^ mice (**B**). Scale bars = 100 μm.

To investigate possible differences of protein expression in the livers of *Smad3* WT and *Smad3* KO mice, the overall protein content in liver tissues of both groups was analyzed by using two-dimensional gel (2-DE) electrophoresis (Figure 3A) and mass spectrometry (MS). As a result, we found that ten proteins were down-regulated and eight proteins were up-regulated in the *Smad3* KO mice compared to the *Smad3* WT mice after four weeks of exposure to CCl_4_ treatment (Table 2). As shown in Figure 4A, glutathione peroxidase, glutathione *S*-transferase theta 2 (GST class-theta 2), NADH-ubiquinone oxidoreductase (subunit B14.7) and peroxiredoxin 6 (antioxidant protein 2) were significantly increased in CCl_4_ treated *Smad3* KO mice. These proteins play an important role in the defense against oxidative stress by reducing reactive oxygen species (ROS). Next, we confirmed the proteomic result by western blotting. *SMP30* was increased in CCl4 treated *Smad3* KO mice, compared to CCl4 treated *Smad3* WT mice (Figure 3B). Also, *SMP30* protein level was elevated in control *Smad3* KO mice, compared to control *Smad3* WT mice (Figure 3C).

### 2.3. Increased Expression of *Smad3* in *SMP30* KO Mice

To confirm the relationship between *Smad3* and *SMP30 in vivo*, we used *SMP30* KO (*SMP30^Y^*^/−^) mice and analyzed phospho-*Smad3* (p-*Smad3*) levels in their livers acutely damaged by CCl_4_ treatment. Immunohistochemical examination revealed that an exposure to CCl_4_ increased p-*Smad3* levels in the livers of *SMP30* KO mice as well as WT mice; however, the *SMP30* KO mice had significantly more p-*Smad3* than the *SMP30* WT mice (Figure 4).

## 3. Discussion

A major finding of the present study is that *Smad3* deficiency significantly prevented CCl_4_-induced liver fibrosis by reducing antioxidant protein expression as well as blocking TGF-β signaling in the liver. Treatment of *Smad3* KO mice with CCl_4_ for 4 weeks successfully induced moderate liver fibrosis. Findings from the histological analysis correlated with data from immunohistochemical testing in which α-SMA was used as a fibrotic marker. All of these data suggested that the absence of *Smad3* resulted in reduced hepatic damage due to CCl_4_ treatment. On the other hand, high levels of calcium deposition were observed in the centrilobular areas of *Smad3* KO mice, indicating that the hepatocytes had sustained severe damage. Haschek and Rousseaux demonstrated that increased intracellular calcium contents exacerbate cellular damage, resulting in hepatic fibrosis [12]. Interestingly, CCl_4_-treated *Smad3* KO mice in the present study had more calcium deposition than CCl_4_-treated WT animals, but less fibrosis was found in the CCl_4_-treated *Smad3* KO mice. This is not consistent with findings from the study by Haschek and Rousseaux. We questioned why *Smad3* deficiency resulted in less fibrosis even with significant calcium deposition. So far, we have not identified a clear reason for this result. We assumed that the high levels of calcium deposition in CCl_4_-treated *Smad3* KO mice may not be related to severe cell damage because 1) an increase in PCNA-positive signals (usually detected during active cell proliferation) was found in the CCl_4_-treated *Smad3* KO mice, and 2) up-regulation of cell protective gene expression was identified during proteomic analysis.

Proteomic evaluation demonstrated that CCl_4_-treated *Smad3* KO mice had increased expression of antioxidant-related proteins such as *SMP30*, SP56, and GST. In particular, *SMP30* expression was markedly up-regulated in *Smad3* KO mice (Table S1), compared to WT mice. *SMP30* is a major senescence marker and its expression is reduced during the aging process [13], *SMP30* is also involved in the vitamin C synthesis pathway; *SMP30* KO mice cannot synthesize vitamin C *in vivo* [14]. In a recent report, Son et al. demonstrated that *SMP30* exerts a potent anti-oxidative effect and protects neural cells from oxidative damage [15]. Thus, we assumed that deletion of the *Smad3* protein triggered an increase of *SMP30* protein expression, resulting in the prevention of hepatic cell death due to CCl_4_-induced injury. Although further studies are required to determine whether *SMP30* is a key protein that directly prevented liver fibrosis in the present study, the fact that *Smad3* deficiency led to up-regulated *SMP30* expression supports the speculation that *SMP30* expression may play an opposing role to that of *Smad3* during the induction of hepatic fibrosis through an unknown mechanism.

The phenomenon was confirmed by another set of animal experiments with *SMP30* KO mice, indicating that SMP30 deficiency increases the levels of phospho-*Smad3* which is a key protein to promote liver fibrosis. To investigate the precise role of *SMP30*, we evaluated male *SMP30* KO mice with acute CCl_4_-induced hepatotoxicity. After CCl_4_-induced injury, the levels of phospho-*Smad3* in the *SMP30* KO mice were significantly higher than those in the *SMP30* WT littermates. Enhanced expression of phospho-*Smad3* in the *SMP30* KO mice we observed concurred with our finding of decreased hepatic fibrogenesis in *Smad3* KO mice. In general, the phosphorylation of *Smad3*, a key modulator of the TGF-β1 signaling pathway, is induced during hepatic fibrogenesis [2].

The expression of glutathione peroxidase, GST theta, NADH-ubiquinone oxidoreductase, and peroxiredoxin 6 (antioxidant protein 2) was also up-regulated in *Smad3* KO mice (data not shown). These proteins are involved in metabolism and are associated with the ROS defense systems [16]. In particular, peroxiredoxin 6 has been known as as an acidic calcium-independent phospholipase A2 and a non-selenium glutathione peroxidase. Since proteins related to the antioxidant system were more highly expressed in *Smad3* KO mice than in the *Smad3* WT mice after CCl_4_ treatment, their presence presumably provided a significant level of protection against hepatic fibrosis.

## 4. Material and Methods

### 4.1. Animals

*Smad3* KO mice generated by targeted disruption of the *Smad3* gene via homologous recombination were kindly provided by Anita B Robert (National Cancer Institute, Bethesda, MD, USA). These mutant mice were housed in a room at 22 ± 2 °C, with a 12-h light-dark cycle and were given food and water *ad libitum*. Genotypes of *Smad3* mutant mice were determined using tail DNA (Figure S1A). For PCR analysis, the *Smad3* WT allele was detected using primer 1: 5′-CCACTTCATTGCCATATGCCCTG-3′ and primer 2: 5′-CCCGAACAGTTGGATTCACACA-3′. This primer pair amplifies a fragment of 431 bp from *Smad3* WT mice, but not from *Smad3* KO mice. DNA was also amplified using the primer 1 and primer 3: 5′-CCAGACTGCCTTGGGAAAAGC-3′, which is located in the pLoxpneo to detect the mutant *Smad3* allele. In this case, a 284 bp fragment was detected for the *Smad3* KO allele, whereas no such signal was detected in the wild-type mice [17]. *Smad3* KO mice were previously generated with the gene targeting technique and kindly provided by Akihito Ishigami in Tokyo Metropolitan Institute of Gerontology (Tokyo, Japan) and heterozygous female mice (*SMP30*^+/−^) were mated with male knockout mice (*SMP30^Y^*^/−^) to produce male knockout (*SMP30^Y^*^/−^) and male wild type (*SMP30^Y^*^/+^) littermates. Genotypes of SMP30 mutant mice were determined as described previously (Figure S1B) [18].

### 4.2. Treatment of Animals

Six-week-old male *Smad3* WT and *Smad3* KO mice were divided into four groups: *Smad3* WT, *Smad3* KO, CCl_4_-treated *Smad3* WT, and CCl_4_-treated *Smad3* KO mice. In the CCl_4_-treated animals, liver fibrosis was induced by intraperitoneal injection of 2 mL/kg of CCl_4_ (Wako Pure Chemical Industries, Osaka, Japan) three times a week for 4 weeks. The mice were sacrificed 24 h after the last injection. For the induction of acute liver injury, *SMP30^Y^*^/+^ and *SMP30^Y^*^/−^ mice were injected intraperitoneally with CCl_4_ every 2 days for a week. Twenty-four hours after the third CCl_4_ injection, liver tissues were collected from all mice.

### 4.3. Histopathological Evaluation of the Liver Tissue

Pieces of liver were rapidly fixed in 10% neutral buffered formalin and embedded in paraffin. The sections were stained with hematoxylin and eosin (H & E), and azan. The severity of hepatic fibrosis was graded on a scale of 0–4 as previously reported [11]. Calcium deposition in liver tissues from the CCl_4_-treated groups was evaluated using von Kossa stain. For immunohistochemistry, anti-PCNA (Santa Cruz Biotechnology, Santa Cruz, CA, USA), anti-α-SMA (Sigma, St. Louis, MO, USA), and anti-phosphorylated (phospho)-*Smad3* (Cell Signaling Technology, Danvers, MA, USA) antibodies were used as primary antibodies. Antigen-antibody complexes were visualized by using an avidin-biotin peroxidase complex solution and an ABC kit (Vector Laboratories, Burlingame, CA, USA) with 3,3-diamino benzidine (Zymed Laboratories Inc., South San Francisco, CA, USA).

### 4.4. Preparation of Liver Tissue for Proteomic Analysis

Frozen liver tissue (200 mg) was homogenized in lysis buffer (40 mM Tris, 8 M urea, 4% CHAPS, 1 mM EDTA, and 10 mM dithioerythritol). The protein concentration of the homogenates was estimated using a commercial 2-D Quant Kit (Amersham Biosciences, Uppsala, Sweden) [19].

### 4.5. Two-Dimensional Gel Electrophoresis and Data Processing

A first-dimensional isoelectric focusing (IEF) experiment was carried out on commercial 18 cm, immobilized pH gradient (IPG) strips (pH 3–10), in an IPGphor electrophoretic unit (Amersham Biosciences, Pittsburgh, PA, USA). The second-dimension was separated on 12% SDS-polyacrylamide gel electrophoresis (SDS-PAGE) at 15 °C in an Ettan DALTsix apparatus (Amersham Biosciences, Pittsburgh, PA, USA). All samples were run in triplicate to ensure reproducibility of results. After fixation, the gels were stained with Coomassie blue (Bio Basic Inc., Markham, ON, Canada). Spot intensities in gel image were analyzed with PDQuest 2D Analysis Software (BioRad, Hercules, CA, USA). Statistical analysis of the spot intensities was performed by Student’s *t-*test (*p* < 0.05). The significance of level was taken more than two-fold changes of volume percentage [20].

### 4.6. Matrix-Assisted Laser Desorption/Ionization Time of Flight Mass Spectroscopy (MALDI-TOF/MS)

Targeted spots were excised, and an in-gel digestion was performed. Briefly, enzymatic digestion was performed by adding modified trypsin (Promega, Madison, WI, USA) in 50 mM ammonium bicarbonate and 5 mM calcium chloride then incubation at 37 °C for 16 h. These spots were analyzed by using MALDI-TOF/MS (Voyager-DE STR, Applied Biosystems, Foster, CA, USA). A database search was then conducted with the MS-Fit (http://prospector.ucsf.edu) to locate possible matches.

### 4.7. Immunoblotting

Snap-frozen liver tissues were homogenized in a RIPA buffer containing 0.1 mM of Na_3_VO_4_ and Protease Inhibitor Cocktail Tablets (Roche, Mannheim, Germany). Protein samples (30 μg per lane) were separated by 10% SDS-PAGE and electro-transferred to a PVDF membrane (Schleicher & Schuell, Dassel, Germany). The primary antibodies used were anti-*SMP30* antibody (SantaCruz Biothechnology, Dallas, TX, USA), and anti-β-actin antibody (SantaCruz Biotechnology, Dallas, TX, USA). Specific binding was detected by using the Super Signal West Dura Extended Duration Substrate (Pierce, Rockford, IL, USA) and by exposure of the blots to Medical X-ray Film (Kodak, Tokyo, Japan).

### 4.8. Statistical Analysis

The results are expressed as the means ± S.D. To compare values obtained from the two groups, Student *t* test was done. A value of *p* < 0.05 was considered significant.

## 5. Conclusions

Results from the current investigation indicated that *Smad3* likely participates in the antioxidant defense system by regulating the production of antioxidant proteins such as *SMP30* that protects against liver injury and mediates TGF-β signaling. A more depth-in study will be required to further elucidate the underlying mechanism. Based on our findings, we suggest that the *Smad3* may play a critical role in hepatic fibrosis development mediated by the regulation of antioxidant gene expression and TGF-β signaling.

## Supplementary Information



## Figures and Tables

**Table 1.** Hepatic lesions and grade of fibrosis in the livers of *Smad3*-mutant mice after the CCl_4_-treatment.

Animal treatment (number of animals)	Hepatic lesions after CCl_4_-treatment	Grade A	α-SMA B
*Smad3^WT^* mice (*n* = 5)	Normal	0.10 ± 0.22	0.74 ± 0.25
*Smad3^KO^* mice (*n* = 5)	Enlargement of hepatocyte	0.08 ± 0.18	0.68 ± 0.22
CCl_4_-treated *Smad3^WT^* mice (*n* = 5)	Pseudolobule formation, Degenerative and necrotic cells	2.60 ± 0.47 *	2.40 ± 0.47 *
CCl_4_-treated *Smad3^KO^* mice (*n* = 5)	Bridging fibrosis, Severe calcium deposition	1.40 ± 0.55 *	1.84 ± 0.49 *

A Grade of hepatic fibrosis—0, none; 1, short collagenous septa extended from central veins; 2, slender septa link the central veins but lobular architecture is preserved; 3, pseudolobuli are formed by thin septa; 4, parenchyma is subdivided into smaller pseudolobuli by thin septa. Liver fibrosis was graded based on previously reported scoring system [11];

B Immunohistochemical expression for α-smooth muscle actin—0, none; 1, mild; 2, moderate; 3, severe expression. Data are shown in Figure 2C;

* Significant differences in *Smad3^WT^* and *Smad3^KO^* mice (Student’s *t* test, *p* value < 0.005).

**Table 2.** List of notably changed proteins in *Smad3* KO mice liver after CCl_4_ treatment.

Spot No.	Protein Name	Accession No. a	MOWSE Score b	Masses Matched (%)	MW (kDa)	pI c	Relative ratios d
**Down-regulated proteins in CCl_4_-treated KO mice compared to CCl_4_-treated WT mice**							

54	Rho guanine nucleotide exchange factor 7	Q9ES28	3.32 × 10^4^	17	80	6.50	0.30
55	78 kDa glucose-regulated protein precursor	P20029	1.48 × 10^7^	36	72	5.10	0.21
56	Heat shock cognate 71 kDa protein (HSP 70)	P63017	7.85 × 10^9^	44	70	5.40	0.36 *
57	Leucine-rich repeat LGI family member 4 precursor	Q8K1S1	1.46 × 10^4^	10	59	7.90	0.19
59	Adenosylhomocysteinase (Liver copper binding protein)	P50247	7.03 × 10^4^	13	48	6.10	0.18
60	Neutrophil cytosol factor 1 (NCF-1)	Q09014	1.25 × 10^4^	12	45	9.10	0.15
61	Wnt-11 protein precursor	P48615	1.77 × 10^4^	15	39	9.10	0.27
62	Uricase (Urate oxidase)	P35688	1.33 × 10^4^	21	35	8.50	0.24
63	Thioether *S*-methyltransferase (TEMT)	P40936	1.96 × 10^5^	15	30	6.00	0.43
64	Ferritin light chain 1 (Ferritin L subunit 1)	P29391	9.28 × 10^5^	22	21	5.70	

**Up-regulated proteins in CCl4-treated KO mice compared to CCl4-treated WT mice**							

19	Selenium-binding protein 1(SP56)	P17563	1.24 × 10^4^	18	52	6.00	2.86 *
21	Senescence marker protein-30 (*SMP30*)	Q64374	3.55 × 10^6^	22	33	5.20	2.58 *
22, 23	Glutathione *S*-transferase Mu 1	P10649	1.29 × 10^6^	21	26	7.70	2.61 *
65	Glutathione peroxidase (Cellular glutathione peroxidase)	P11352	8.77 × 10^3^	10	22	6.70	5.07
67	Glutathione *S*-transferase theta 2 (GST class-theta 2)	Q61133	1.12 × 10^4^	7	28	7.00	
66	NADH-ubiquinone oxidoreductase subunit B14.7	Q9D8B4	4.40 × 10^3^	10	15	8.60	12.15
68	Peroxiredoxin 6	O08709	5.65 × 10^5^	19	25	5.70	
69	Alpha-1-acid glycoprotein 1 precursor	Q60590	2.60 × 10^4^	10	24	5.60	2.39

a Accession No.: Protein No. of SwissProt database;

b MOWSE score: Based on the number of peptides matching the protein in the database and the accuracy of those matches;

c MW and pI: Obtained from the MS fit search of proteinprospector database;

d Relative ratio: Relative % volume of spot in KO compared to WT (1>, up-regulation in knockout type; 1<, down-regulation in knockout type);

* Significant differences in the *Smad3^WT^* mice (Student’s *t* test, *p* value < 0.05).
