# Supplementary material for: Smad3 Deficiency Ameliorates Hepatic Fibrogenesis through the Expression of Senescence Marker Protein-30, an Antioxidant-Related Protein
Source: Int J Mol Sci. 2013 Dec 4;14(12):23700–10. doi: 10.3390/ijms141223700 (PMC3876072; doi:10.3390/ijms141223700)
Supplement: Supplementary file 1 [file ijms-14-23700-s001.pdf]

Supplementary Information

Figure S1. Confirmation of genotype in *Smad3* and *SMP30* mutant mice. (**A**) Ethidium bromide-stained agarose gel showing polymerase chain reaction (PCR) products of tail DNA from *Smad3* mutant mice. The 431 bp band (lane 1) indicates the wild-type (*Smad3*$^{+/+}$) and the 284 bp band (lane 4) represents knockout (*Smad3*$^{-/-}$) mice. Lane designations: lane M, DNA molecular size marker; lane 1, tail DNA of *Smad3*$^{+/+}$ mice with primer 1 plus primer 2 mixture; lane 2, tail DNA of *Smad3*$^{+/+}$ mice with primer 1 plus primer 3 mixture, lane 3, tail DNA of *Smad3*$^{-/-}$ mice with primer 1 plus primer 2 mixture, lane 4, tail DNA of *Smad3*$^{-/-}$ mice with primer 1 plus primer 3 mixture; and (**B**) Ethidium bromide-stained agarose gel showing PCR products of of tail DNA from *SMP30* mutant mice. The *SMP30*$^{Y/+}$ and *SMP30*$^{Y/-}$ genes gave 280 bp and 1363 bp PCR products, respectively.

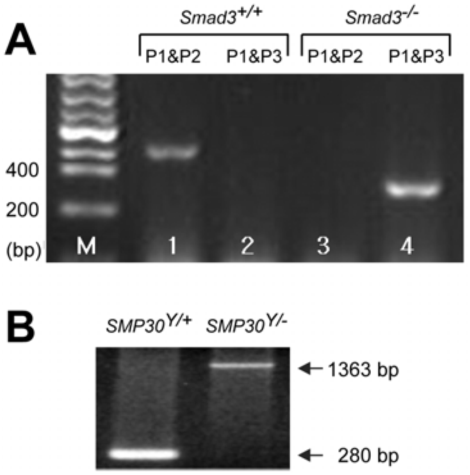

Table S1. Markedly changed hepatic proteins in *Smad3*$^{-/-}$ mice *vs.* *Smad3*$^{+/+}$ mice.

Spot No.	Protein Name	Accession No. [A]	MOWSE Score [B]	Masses Matched (%)	MW [C] (kDa)	pI [C]	Relative ratios [D]
	Down-regulated proteins in livers of *Smad3*$^{-/-}$ mice						
1	Semaphorin 3E precursor	P70275	9.84×10^4	21	90	8.00	0.00
2	Probable urocanate hydratase	Q8VC12	2.87×10^3	18	75	7.30	0.34
3	Heat shock cognate 71 kDa protein (HSP 70)	P63017	7.85×10^9	44	70	5.40	0.36 *
4	Nonspecific lipid-transfer protein	P32020	1.23×10^4	13	59	7.20	0.00
5	Ubiquinol-cytochrome-c reductase complex core protein 2	Q9DB77	7.42×10^3	14	48	9.30	0.00 *
6	T-cell ectoADPribosy-transferase 1 precursor (T-cell differentiation marker Rt6 homolog 1)	P17981	3.32×10^3	11	32	8.40	0.34 *
	Up-regulated proteins in livers of *Smad3*$^{-/-}$ mice						
18	Perilipin (Lipid droplet-associated protein)	Q8CGN5	4.15×10^3	20	56	6.60	2.20
19	Selenium-binding protein 1(SP56)	P17563	1.24×10^4	18	52	6.00	2.86 *
21	Senescence marker protein-30 (*SMP30*)	Q64374	3.55×10^6	22	33	5.20	2.58 *
22,23	Glutathione S-transferase Mu 1	P10649	1.29×10^6	21	26	7.70	2.61 *

[A] Accession No.: Protein No. of SwissProt database (2007.04.19); [B] MOWSE score: Based on the number of peptides matching the protein in the database and the accuracy of those matches; [C] MW and pI: Obtained from the MS fit search of proteinprospector database; [D] Relative ratio: Relative % volume of spot density in *Smad3*$^{-/-}$ compared to *Smad3*$^{+/+}$ mice (1>, up-regulation in *Smad3*$^{-/-}$ mice; 1<, down-regulation in *Smad3*$^{-/-}$ mice); * Significant differences in the *Smad3*$^{+/+}$ mice (Student's *t* test, p value <0.05).
